# Microbial engineering for pesticide degradation: current insights and future directions for sustainable agriculture

**DOI:** 10.3389/fmicb.2026.1751932

**Published:** 2026-02-23

**Authors:** Sudhakar Srivastava, Rakeeb Ahmad Mir, Sofi Javed Hussain, Suchitra Mitra, Shruti Srivastava, Pankaj Kumar, Harmanjit Kaur

**Affiliations:** 1 Institute of Environment and Sustainable Development, Banaras Hindu University, Varanasi, India; 2 Department of Biotechnology, School of Life Sciences, Central University of Kashmir, Ganderbal, India; 3 Department of Botany, Central University of Kashmir, Ganderbal, India; 4 Indian Institute of Science Education and Research, Kolkata, India; 5 Department of Botany and Microbiology, H.N.B. Garhwal University (A Central University), Srinagar Garhwal, India; 6 Department of Botany, University of Allahabad, Prayagraj, India

**Keywords:** bio-remediation, degradation, microbiome engineering, pesticides, rhizosphere

## Abstract

Pesticides are synthetic agrochemicals widely used to protect crops from pests and diseases; however, their limited biodegradability and indiscriminate application pose serious risks to non-target organisms, soil fertility, human health, and overall environmental sustainability. Conventional physical and chemical remediation strategies often fall short in restoring contaminated ecosystems, highlighting the urgent need for effective and sustainable pesticide mitigation approaches. In recent years, *in situ* bioremediation has emerged as a promising, eco-friendly, and cost-effective strategy for pesticide degradation in agricultural soils. Under favourable conditions, microorganisms utilise pesticides as sources of carbon, sulphur, and electrons, facilitating their breakdown through diverse metabolic pathways, with enzymatic degradation playing a central role in chemical transformation. Microbial consortia exhibit enhanced degradation efficiency by leveraging functional diversity and synergistic interactions among their microbial members. For instance, a consortium comprising *Azospirillum*, *Cloacibacterium*, and *Ochrobacterium* achieved 100% degradation of 50 mg L^−1^ glyphosate within 36 h. Advances in microbiome engineering have further expanded the scope of bioremediation by enabling the targeted manipulation of microbial communities to improve degradation specificity and performance. Notably, the recombined genomes of *Psathyrella candolleana* and *Pseudomonas putida*, generated through protoplast fusion, degraded 78.98% of pentachlorophenol in contaminated water. Additionally, engineering the rhizosphere with plant growth–promoting microorganisms, combined with microbial genetic modification, has demonstrated significant potential in enhancing pesticide degradation while simultaneously improving crop growth and productivity. Such integrative approaches represent a sustainable pathway towards resilient agroecosystems. This review synthesises current knowledge on the impacts of pesticides on crop physiology and metabolism, explores conventional and advanced microbe-mediated degradation strategies, and highlights the role of microbial engineering and consortia-based systems. Furthermore, it discusses emerging technologies, environmental and economic benefits, and recent patentable innovations, underscoring their relevance for sustainable agriculture and ecological restoration.

## 1 Introduction

Pesticides are industrially synthesized chemical compounds, which are broadly applied to agricultural crops to protect them from pests (Zhou et al., 2025). A range of pesticides, for example, organochlorines, organophosphates, carbamates, pyrethroids etc. are frequently used to manage or lessen pest load in agricultural systems (Lin et al., 2020; Zhan et al., 2020). Pesticides are in use in agriculture since several years, and the earliest pesticide to be synthesized was dichloro-diphenyl-trichloroethane (DDT). After twenty years of its invention, its use was prohibited in agricultural ecosystems (Egbuna and Sawicka, 2020). Ever since then, numerous other pesticides have been introduced in the market globally, majority of them claiming to be harmless. The application of pesticides has increased dramatically to ensure adequate food for an ever-expanding population (Aryal and Aryal, 2023). India, where the greater part of the population is reliant on agriculture, has experienced a considerable increase in pesticide consumption over time (Kashyap et al., 2024). In 2023, the gross amount of pesticides employed in the farming sector globally was around 3.728 million tonnes, with an average application to crops around 2.40 kg/ha (FAOSTAT, 2023). Out of 3.728 million tonnes of total pesticides used, herbicides constituted approximately 2.009, insecticides 0.759, fungicides and bactericides 0.778, rodenticides 0.017 and other pesticides 0.114 million tonnes (FAOSTAT, 2023). Brazil, United States, Indonesia, Argentina, China, Australia, Vietnam, Russia Federation, Canada and France are the top 10 countries in agricultural consumption of pesticides worldwide (Sasu, 2025). There are certain pesticides whose products have either deteriorated, been banned, or were procured in excess, and hence they are no longer in use (called obsolete pesticides). The total quantity of such pesticides in developing countries is around 400,000–500,000 tonnes. For example, use of DDT is banned worldwide under the Stockholm Convention but in India, its restricted use for public health programs (e.g., malaria control) is allowed up to 10,000 metric tonnes yearly under stringent governmental administration. Similarly, a study reported that approximately 146,000 tonnes of pesticides were banned in European Union but they were being used in United States in 2016 (Donley, 2019). In 2024, nearly 122,000 tonnes of banned pesticides were permitted for export from the European Union, including 1, 3-dichloropropene: over 20,000 tonnes, Glufosinate: almost 20,000 tonnes and Mancozeb: more than 8,500 tonnes (Fleck, 2025).

Nearly ≤5% of the total used pesticides inhibits the targeted pest organisms, whereas left over >95% of these pesticides do not reach the designated pests. Subsequently, the remains of the pesticides are accumulated in the adjacent environments, get mixed with water and soil and contaminate them (Sarker et al., 2021). Pesticide remains have damaging impacts on diverse life forms and ecosystems (Yadav and Devi, 2017) because of their persistent and bio-magnification properties. Pesticides frequently pollute water, soil and air, leading to long-lasting damage to environment and a major threat to human health (Rani et al., 2021; Mukherjee et al., 2024), even in minute traces. Approximately 2.2 million people, generally from developing countries, are at larger risk from pesticide exposure (Kaur et al., 2019). According to World Health Organization (WHO), pesticides are accountable for roughly three million people suffering from poisoning and 2,00,000 deaths yearly (López-Benítez et al., 2024). Furthermore, rigorous use of pesticides has caused surface and groundwater pollution (Dhankhar and Kumar, 2023), ensuing from agricultural runoff, with their subsequent percolation into the soil (Sharma et al., 2019a), as well as contaminating the marine ecosystem. Pesticides are also harmful for the plants (Dreistadt, 2016) and get distributed via food webs (Kumar et al., 2021a). Several regulations/guidelines have been recommended to evaluate human health risks and environmental effects emanating from the use of extremely lethal and persistent pesticides, to restrain their market value, and to ensure proper control of their residues. International organizations, for instance, World Health Organization (WHO), Food and Agriculture Organization (FAO) and Environmental Protection Agency (EPA) have established legal guidelines for pesticide regulation (Naidenko, 2020). In spite of these regulatory attempts, pesticide pollution remains a universal challenge, especially for developing nations, where pesticide use is continuously rising without proper regulation (Zikankuba et al., 2019). Therefore, developing potent and sustainable means for pesticide degradation is important for mitigation of their harmful impacts and protection of natural resources.

Various methods of remediation have been developed, which are largely based on the type and class of pollutants (Karimi et al., 2022; Mahalle et al., 2025). Pesticides present in the soil can be degraded by several ways; the conventional methods include physical, chemical, and physio-chemical degradation, which mostly result in secondary pollution (Karimi et al., 2022). Bioremediation is a broadly recognized, environment-friendly, and sustainable method of depolluting a contaminated environment (Bokade et al., 2021). Microorganisms have great resilience, biochemical flexibility, functional diversity, and employ diverse kinds of metabolisms to degrade pesticides, which they use as source of nitrogen, carbon, phosphorus and energy (Kumar et al., 2021b; Rodríguez et al., 2020). In general, microbes metabolize pesticides in two ways, (1) complete breakdown of the compounds or (2) mineralization of pesticides, in which majority of the by-products are fit for release into the environment (Dar et al., 2022). Additionally, biodegradation of pesticides is less costly than traditional approaches, which makes it economically feasible, and the by-products released are almost harmless to the environment (Carles et al., 2021; Dar et al., 2022). According to the US Environmental Protection Agency (USEPA), bioremediation is a useful and eco-friendly approach for restoring polluted environments and boosting sustainable development (Kour et al., 2022). Diverse microorganisms have an inherent capability to degrade pesticides, including bacteria (*Bacillus, Pseudomonas, Arthrobacter, Acinetobacter, Serratia*), actinomycetes (*Streptomyces*), archaea (*Sulfolobus, Methanobacterium*), fungi (*Aspergillus, Penicillium, Trichoderma*), and algae (*Chlorella, Chlamydomonas*) (Guerrero Ramírez et al., 2023; Aparici-Carratalá et al., 2023; Dinakarkumar et al., 2024; Akmukhanova et al., 2025). Nevertheless, the slow efficiency of these microbes, coupled with a complex and unstable natural environment, may influence the viability and efficiency of microbe-mediated degradation of pesticides. Therefore, there is a dire need to create genetically engineered microorganisms (GEMs) to increase the production of genes and their products with an aim to enhance their pesticide degradation potential.

Several reviews highlighting the potential use of microorganisms in pesticide degradation are available in the literature. However, a review on the genetic manipulation of microbes to increase their pesticide degradation capability in agricultural soil ecosystems has not been performed. Such a review would aid in identifying the potential of microbial engineering technology and examine the existing information, thereby assisting in the development of effective remediation approaches for agricultural soils. This review aimed to gather and analyze scientific research conducted on the use of genetic engineering for producing GEMs, along with the advantages of using microbial consortia as an alternative to conventional technologies for the degradation of pesticides in agricultural soils. The examination of the collected literature facilitated exploring whether the use of GEMs is financially feasible to improve the efficiency of pesticide degradation. This review provides a holistic perspective on the impacts of pesticides on growth and metabolism of crop plants, conventional approaches employed for microbe-mediated degradation of pesticides, how genetic manipulation in microbes can be efficiently utilized in pesticide bioremediation, benefits of using microbial consortia for pesticide degradation, advantages of microbial engineering for environment and economic benefits, thereby identifying new research opportunities and establishing novel practical applications. To achieve the aim, a comprehensive meta-analysis of relevant literature was conducted. We focused on microbiome engineering, microbial pesticides, rhizosphere and patents related to microbial pesticides and their biodegradation properties. The primary databases used for searching literature included Web of Science, PubMed, Scopus, and others. This approach enabled a structured evaluation of existing knowledge, while also highlighting opportunities for future research and innovation.

## 2 Effect of pesticides on growth and metabolism of crop plants

Pesticides can deliver agronomic benefits when used in appropriate amounts. Seed treatments suppress early seed- and soil-borne pests and pathogens, improve seedling emergence and establishment, and grain yield in winter wheat under real field conditions (Turkington et al., 2016). Strobilurin fungicides have also been shown to improve nitrogen use efficiency and support yield and protein targets in durum wheat grown under rainfed Mediterranean conditions, consistent with better maintenance of green area and delayed senescence reported for this fungicide class (Carucci et al., 2020). The defence activator acibenzolar-S-methyl (ASM) primes systemic acquired resistance and can reduce disease severity and spray intensity, an effect confirmed in *Arabidopsis* and reviewed across crops (Ito et al., 2024). Herbicide safeners have been reported to assist crops in tolerating herbicides by inducing detoxification pathways such as glutathione S-transferases (GSTs), UDP-glycosyltransferases (UGTs), and ABC transporters, thereby reducing crop injury (Dimaano and Iwakami, 2021; Deng, 2022). However, pesticide residues in harvested commodities introduce a food-safety dimension. Although processing steps, such as washing, peeling, and thermal treatment, cause reduction in residues (Kim et al., 2017; García-Vara et al., 2022), still they may be present in alarming levels. Current European surveillance indicated that 3.9% of 87,863 samples exceeded maximum residue limits in 2021 and 3.7% of 110,829 samples in 2022 (EFSA et al., 2023, 2024).

**Figure 1:**
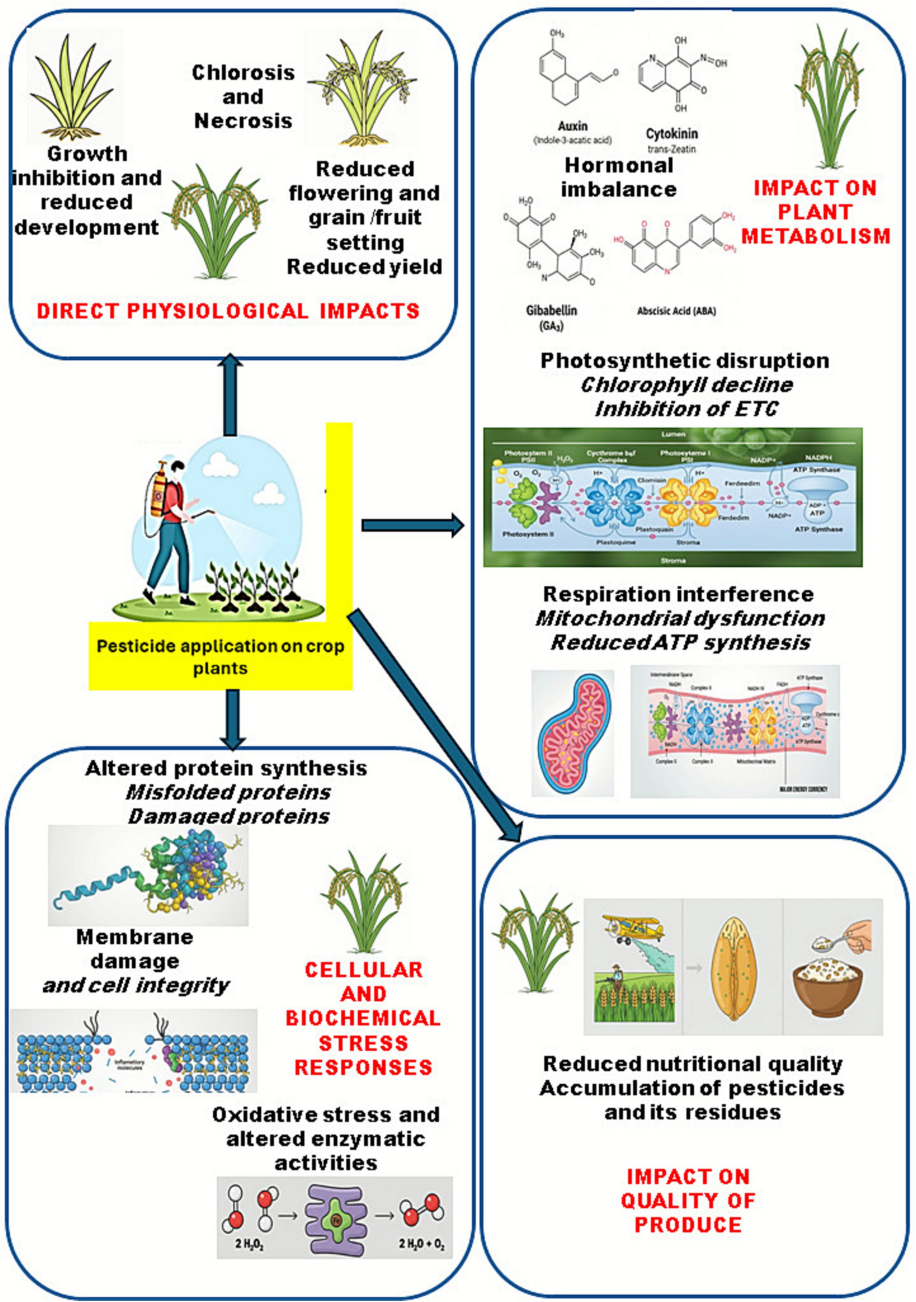
Mechanistic overview of pesticide effects on plant physiology and productivity. The effects of pesticides range from biochemical changes, metabolic perturbations, physiological modifications to ultimate changes in yield and product quality. The pictorial representation demonstrates that it is the overall effects on oxidative metabolism, enzyme activities, hormone levels, photosynthetic and respiratory metabolisms, altered root, shoot and leaf growth and flowering and produce development that results into loss of yield and quality of plant products.

Despite their positive role, pesticides can have negative effects on plant growth and metabolism when applied incorrectly or in excessive amounts (Alengebawy et al., 2021) (Table 1; Figure 1). Studies have documented reduced biomass, altered phenology and architecture, visible injury such as chlorosis and necrosis, and hormonal disruptions affecting auxins, cytokinins, gibberellins, ethylene, and abscisic acid in response to excess pesticide applications (Sharma et al., 2019b; Mukherjee et al., 2022; Guedes et al., 2023; Virk et al., 2024). Mechanistically, many pesticides impair photosynthetic machinery, reduce pigment pools, and trigger oxidative stress that damages membranes and disrupts carbon and nitrogen metabolism (Zhang et al., 2019; Hasanuzzaman et al., 2020; Hatamleh et al., 2022; Traxler et al., 2023). Experimental work demonstrates that chlorophyll fluorescence (PSII) and chlorophyll content can decline after neonicotinoid exposure in crops and seedlings, resulting in associated yield penalties, as observed in long-term studies in chickpea and lettuce (Liu et al., 2021; Shahid et al., 2021). These physiological changes are consistent with earlier canopy senescence and reduced photosynthetic capacity under stress (Hasanuzzaman et al., 2020). Organophosphates exemplify redox-driven phytotoxicity. In rice, sub-chronic chlorpyrifos exposure perturbed physiology and induced oxidative stress (Mu et al., 2022). In maize also, chlorpyrifos caused toxicity, however, soil amendments such as biochar and compost mitigated pigment loss, membrane damage, and oxidative markers (Aziz et al., 2021). These effects lead to yield loses through smaller or prematurely senescing canopies and impaired photochemistry that lower radiation-use efficiency. Beyond direct plant effects, pesticides also reshape soil biology. Studies have reported reductions in microbial biomass and shifts in community structure, as well as suppression of soil enzymes, including dehydrogenases, *β*-glucosidases, and phosphatases, which regulate nutrient cycling and rhizosphere signalling (Walder et al., 2022; Hou et al., 2011; Ghosh et al., 2023; Daunoras et al., 2024). Table 2 summarizes some studies demonstrating the effects of various pesticides on different plants. Thus, while pesticides play a role in crop protection, their adverse impacts on plant growth and metabolism necessitate careful management and adoption of sustainable practices.

**Table 1:** Pesticides and their residues in plants along with their produce and products.

Crop plant	Pesticide treatment details	Residue content in plant parts	References
Durum Wheat (cv. Platone)	Prothioconazole (150 g L^−1^), Benzovindiflupyr (75 g L^−1^), Acetamiprid (200 g L^−1^) on 14 June 2021; Deltamethrin (25 g L^−1^), *λ*-Cyhalothrin (25 g L^−1^) on 21 June 2021 5 × recommended doses	Milling fractions (bran, middlings) concentrated residues (Pf = 2.9–6.8). Aldrin, endrin, methoxychlor, permethrin: high in bran (Pf 1.9–2.5). Malathion, methoxychlor, lindane: 0–2.6 mg/kg in flour Cypermethrin & fenvalerate: bran > middlings > flour	Pelosi et al. (2025)
Rice (*Oryza sativa* L.)	Deltamethrin, Penconazole, Kresoxim-methyl, Cyproconazole, Epoxiconazole Azoxystrobin; artificial contamination (20–50 μg/kg) air-dried 24 h, washed with mineral water.	Bran contained 2.5 × higher residues Kresoxim-methyl: 20.3 ppb in paddy rice samples (34/50 > EU MRL 0.01 mg/kg); 13.7 ppb in brown rice; 5.53 ppb in polished rice. Processing reduced residues by 66.1–74.7% Polishing removed residues by 43.1–67.8%. Washing reduced 22 pesticides (0.53–30.4%); cadusafos 65.8%, propyzamide 39.1%.	Medina et al. (2021) and Carreiró et al. (2024)
Maize (*Zea mays* L.)	16 pesticides detected in 358 field samples in China including Pyraclostrobin, Tebuconazole, Carbendazim, Triadimefon, Chlorpyrifos, Metolachlor, Thiamethoxam	Kernel residues 1.0–175.9 μg/kg. 21.3% of samples contained ≥1 pesticide (max 6). Pyraclostrobin 1.0–94.5 μg/kg (2 > MRL). Tebuconazole 3.2–175.9 μg/kg (none > MRL). Thiamethoxam 5.5–72.7 μg/kg (2 > MRL). Carbendazim ≤25.0 μg/kg, Triadimefon 2.3–4.0 μg/kg, Chlorpyrifos 2.0–7.6 μg/kg.	Wei et al. (2025)
Fieldpea (*Pisum sativum* L.)	Pendimethalin, Imazethapyr, Quizalofop-p-ethyl at recommended rates	Residues in pea grains and soil <0.05 mg/kg (below detection).	Das et al. (2024)
Onion (*Allium cepa* L., dry bulb)	Fluchloralin (1.0 kg a.i./ha), Pendimethalin (0.75 kg a.i./ha), Oxyfluorfen (0.25 kg a.i./ha); Sequential applications of these pesticides (multiple (repeated) applications.)	Residues: Fluchloralin 0.0098 μg/g; Pendimethalin 0.02 μg/g; Oxyfluorfen 0.005 μg/g. Fluchloralin fb Fluchloralin 0.0173 μg/g, Pendimethalin fb Pendimethalin 0.041 μg/g, Oxyfluorfen fb Oxyfluorfen 0.008 μg/g (all <MRL).	Das et al. (2024)
Rapeseed	Imidacloprid, Thiamethoxam, Quizalofop-p-ethyl	Imidacloprid 0.025 mg/kg; Thiamethoxam 0.012–0.634 mg/kg; Quizalofop-p-ethyl 0.011 mg/kg.	Liu N. et al. (2024)
Cabbage	Organochlorine and organophosphate pesticides incl. p,p’-DDE, Aldrin, Chlorfenvinphos, Fenitrothion, Permethrin, Endosulfan sulphate, Deltamethrin, Fenvalerate, Cypermethrin, Methoxychlor, Fonofos, Diazinon.	DDE 0.04 μg/kg; Aldrin 0.01 μg/kg; Chlorfenvinphos <0.01–0.06 μg/kg; Permethrin <0.01–0.15 μg/kg; Endosulfan sulphate 0.05 μg/kg; Deltamethrin <0.01–1.60 μg/kg; Fonofos 0.43 μg/kg.	Fosu et al. (2017)
Goji	Cypermethrin, Dimethoate, Methomyl, Flutriafol, Carbendazim	Cypermethrin 0.021–0.136 mg/kg; Dimethoate 0.0153–0.595 mg/kg; Methomyl 0.0162–0.0518 mg/kg; Flutriafol 0.0098–0.0693 mg/kg; Carbendazim 0.08–0.096 mg/kg.	Chen et al. (2023)
Mint	Azoxystrobin, Bifenthrin, Cypermethrin	Azoxystrobin 0.101–1.44 mg/kg; Bifenthrin 0.00304–0.0657 mg/kg; Cypermethrin 0.0536–0.110 mg/kg.	Zhang et al. (2023)
Tomato	Dimetomorph	0.01 mg/kg.	Bojacá et al. (2013)
Eggplant/Tomato	Cypermethrin	Eggplant Nd–0.13 mg/kg; Tomato 0.02–0.24 mg/kg	Jallow et al. (2017)
Carrot	Metribuzin (300 g a.i./ha PE; 400 g a.i./ha PE).	0.074 ppm and 0.098 ppm at 84 DAA	Kulshrestha and Singh (2001)
Cocoa beans	Chlorpyrifos	0.04 mg/kg.	Okoffo et al. (2017)

Pf = Processing Factors, i.e., specific concentration or dilution factors of pesticide residues in processed food as they are defined in Reg. (EC) No. 396/2005 (European Commission), 2005). MRL, Maximum Residue Limits; Nd, not detected; a.i./ha, active ingredient per hectare, fb, followed by.

**Table 2:** The effects of various pesticides on the physiology and metabolism of plants.

Crop plant	Pesticide name and treatment details in brief	Effects on plants	References
*Vitis vinifera L. × Vitis labrusca L.* (Grapevine)	Acetochlor, Soil, 22,460 g a.i. ha^−1^, 30 days	Increase in superoxide radicals (O^2·-^) and malondialdehyde (MDA) levels; Decrease in ascorbate peroxidase (APOX), catalase (CAT), peroxidase (POD), and superoxide dismutase (SOD) activities in leaves (upper node)	Tan et al. (2012)
*Brassica napus* L. (Rapeseed)	Napropamide, Seedling, 8 mg L^−1^, 5 days	Increase in thiobarbituric acid reactive substances (TBARS) in leaves	Cui et al. (2010)
*Pennisetum americanum* L.	Atrazine, Soil, 10 mg kg^−1^, 38 days	Increase in malondialdehyde (MDA) in shoot and root	Jiang et al. (2016)
*Lactuca sativa* L. (Lettuce)	Alachlor, Hoagland medium, 2 μM, 24 days	Increase in catalase (CAT) and superoxide dismutase (SOD); Decrease in peroxidase (POD) in leaves	Štajner et al. (2003)
*Lactuca sativa* L. (Lettuce)	Imidacloprid and Fenvalerate, 10 mg/L	Under Imidacloprid treatment: decreased iron, arginine, cysteine, homoserine, 4-hydroxyisoleucine, proline, and total amino acids. Under Fenvalerate treatment: increased iron content, reduced flavonoid and vitamin C levels.	Zhang et al. (2022b)
*Brassica juncea* L. (Indian mustard/Chinese mustard)	Imidacloprid, Soil, 300 mg kg^−1^, 80 days	Increase in ascorbate peroxidase (APOX), guaiacol peroxidase (GPOX), glutathione reductase (GR), glutathione S-transferase (GST), and peroxidase (POD) in green pods	Sharma et al. (2016)
*Oryza sativa* L. (Rice)	Imidacloprid, Sand, 0.01%, 12 days	Increase in ascorbate peroxidase (APOX), dehydroascorbate reductase (DHAR), glutathione reductase (GR), monodehydroascorbate reductase (MDHAR), and superoxide dismutase (SOD); Decrease in catalase (CAT) and peroxidase (POD) in seedlings.	Sharma et al. (2013)
*Oryza sativa* L. (Rice)	Diuron, 0.125 mg/L, 0.25 mg/L, 0.5 mg/L, 1.0 mg/L, 2.0 mg/L	Decreased elongation, biomass, and chlorophyll; Increased malondialdehyde (MDA), superoxide dismutase (SOD), peroxidase (POD), glutathione reductase (GR), polyphenol oxidase (PPO), ascorbic acid peroxidase (APX), catalase (CAT), and jasmonic acid (JA); Glutathione (GSH) increased then decreased	Wang et al. (2022)
*Vigna radiata* L. (Mungbean)	Chlorpyrifos	Enhanced rate of proline content and lipid peroxidation; significantly declined glutathione level	Parween et al. (2018)
*Triticum aestivum* L. (Wheat)	Imidacloprid, 100 mg/kg, 200 mg/kg	Decreased jasmonic acid in root and leaf, decreased indole acetic acid in root and leaf, increased abscisic acid in root and leaf, decreased ferulic acid	Li et al. (2023)
*Zea mays* L. (Maize)	Metolachlor, 0.5 mg/L, 1.0 mg/L, 2.0 mg/L, 4.0 mg/L, 8.0 mg/L	Increased malondialdehyde (MDA), ascorbic acid peroxidase (APX), glutathione peroxidase (GPX), and catalase (CAT); Decreased germination, biomass production, and vigor index; Decreased ethyl carbamate50; MDA increased by 26.0 and 48.9% at 1.0 and 2.0 mg/L, respectively	Panfili et al. (2019)
*Trifolium pratense* L., *Lotus corniculatus* L., *Trifolium repens* L., *Cichorium intybus* L.	Glyphosate, 1,440 g a.i./ha	Decreased cumulative number of *Trifolium pratense* L. flowers and *Lotus corniculatus* L. flowers; Increased cumulative number of *Trifolium repens* L. flowers	Strandberg et al. (2021)
*Glycine max* L. Merr. (Soybean)	Deltamethrin, Spray, 0.20%, 10 days	Increase in ascorbate peroxidase (APOX), glutathione reductase (GR), and superoxide dismutase (SOD); Decrease in catalase (CAT) in leaves	Bashir et al. (2007)
*Elodea canadensis* Michx. (Canadian waterweed), *Eleocharis acicularis* L. (Needle spikerush), *Mentha aquatica* L. (Water mint)	Chlorpyrifos, 50 μg/dm^3^, 100 μg/dm^3^, 150 μg/dm^3^	Increased glutathione peroxidase activity; decreased glutathione S-transferase, chlorophyll a, chlorophyll b, and carotene contents	Sobiecka et al. (2022a, 2022b)
*Arabidopsis thaliana (*Arabidopsis*)*	Dichlorprop, 0.1 μM, 0.2 μM, 0.3 μM	Reduced plant growth; increased H₂O₂, jasmonic acid, and salicylic acid levels; abscisic acid decreased initially and then increased at higher concentrations	Chen et al. (2021)

a.i./ha, active ingredient per hectare.

## 3 Traditional approaches for microbial degradation of pesticides

### 3.1 Natural attenuation

Natural attenuation (NA), also referred to as intrinsic bioremediation, is a passive remediation strategy that relies on the innate metabolic activities of indigenous microbial communities to degrade pesticides and other contaminants *in situ* without direct human intervention (Lopes et al., 2022) Figure 2. The effectiveness of NA is influenced by multiple factors, including type and concentration of pesticides present, composition and abundance of native microbial populations, and environmental conditions such as temperature, pH, moisture, and nutrient availability (Gonçalves and Delabona, 2022). Microbial degradation under anaerobic conditions through NA typically involves enzymatic processes such as hydrolysis, oxidation–reduction, and dechlorination. Bacterial strains like *Pseudomonas* spp., *Bacillus* spp., *Sphingomonas* spp., *Xanthomonas* spp., *Stenotrophomona* and *Bacillus cereus* have been found to degrade chlorpyrifos with variable efficiency (Mahalle et al., 2025). Other studies have also documented capability of bacterial and fungal strains to degrade chlorpyrifos residues (Kumar G. et al., 2022). Enzymes involved in pesticide breakdown are sensitive to environmental parameters. For instance, microbial consortia degrading chlorpyrifos in soil exhibited marked decreases in half-life when nutrient and aeration conditions were optimized (Conde-Avila et al., 2021). In addition to microbial metabolism, NA may be enhanced by sorption, volatilization, and diffusion processes, which reduce the active concentrations and availability of pesticides. A recent review highlighted how low-cost sorbents (e.g., biochar, activated carbon) integrated with NA approach can substantially increase removal efficiencies (over 90%) under optimized laboratory conditions (Bala et al., 2022). However, NA has inherent limitations. It is often slow and may result in incomplete degradation and mineralization and may sometimes lead to generation of persistent intermediates (Lopes et al., 2022).

**Figure 2:**
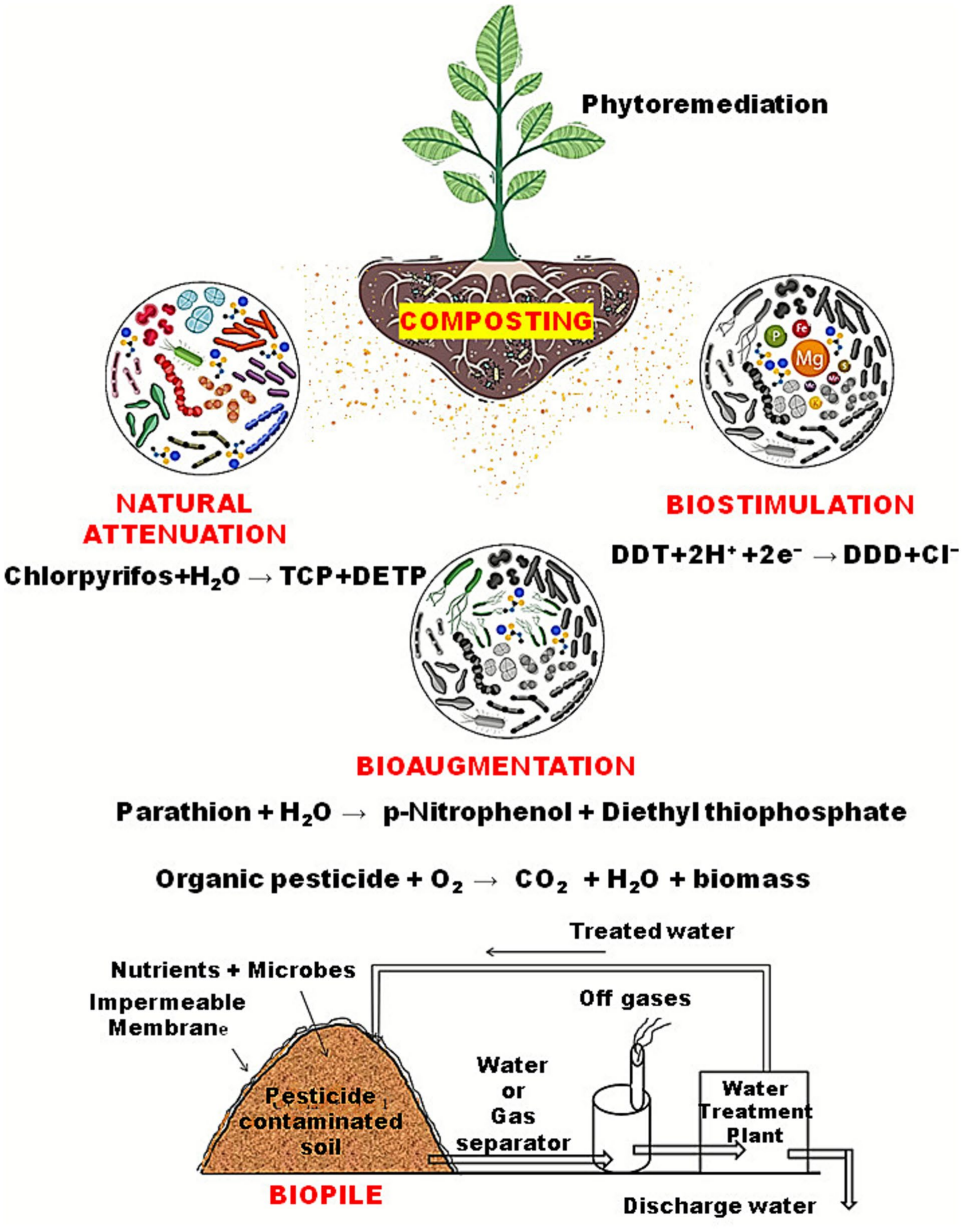
Different methods of degradation of pesticides, i.e., natural attenuation (relying on natural dilution/sorption/biodegrading), bioaugmentation (adding pesticide-degrading microbes to accelerate cleanup), biostimulation (supplying nutrients/oxygen/carbon to boost native degraders), and biopiling (piling and aerating amended soil for controlled biodegrading).

### 3.2 Bioaugmentation

Bioaugmentation is an engineered bioremediation approach that involves introducing specific microbial inoculants to enhance pesticide degradation in contaminated environments (Jia et al., 2021; Bosu et al., 2024) Figure 2. For instance, *Alcaligenes faecalis* DSP3 achieved nearly 100% removal of chlorpyrifos (100 mg kg^1^) in silty clay soil within 12 days, far exceeding the 22% degradation observed in non-bioaugmented controls (Yadav et al., 2021). *Bacillus* spp. are effective pesticide degraders. *Bacillus flexus* XJU-4 aerobically degrades fenvalerate, channeling it through 3-phenoxybenzoate intermediates (Mulla et al., 2017). For pyrethroids, *Bacillus cereus* GW-01 degrades *β*-cypermethrin, and it was found that *in vivo* dosing with *Bacillus cereus* GW-01 reduced β-cypermethrin burdens and toxicity in mice (Zhao et al., 2022; Xie et al., 2023). Other Gram-positives (e.g., *Brevibacillus parabrevis* BCP-09) also remove deltamethrin efficiently (Zhang et al., 2024a). Apart from *Bacillus*, *Serratia marcescens* has demonstrated organophosphate detoxification through microbial degradation in an insect host model, underscoring its enzymatic potential for detoxifying organophosphates (Xia et al., 2023). Fungal systems such as Brown-rot *Fomitopsis pinicola* alone removes a substantial fraction of DDT within days, and co-cultures with bacteria (e.g., *Ralstonia pickettii*) can raise DDT removal to ~60% in lab media (Purnomo et al., 2020). Similar DDT-degrading performance by mixed cultures and other white-rot systems has been reported (Sariwati et al., 2017; Rizqi et al., 2023). Mechanistically, organophosphate hydrolases (OPH/phosphotriesterases) catalyze hydrolysis of P–O/P–S/P–F bonds in organophosphates. Recent work also links OPH to iron-responsive regulation and reiterates that opd loci occur on mobile elements and plasmids that facilitate horizontal transfer (Nandavaram et al., 2023). Finally, microbial consortia often outperform single strains due to metabolic complementarity and ecological stability, a pattern highlighted in recent syntheses (Alidoosti et al., 2024).

### 3.3 Biostimulation

Biostimulation is a widely used approach in bioremediation that enhances the indigenous microbial degradation of pesticides by optimizing environmental conditions (Aldas-Vargas et al., 2021). This method involves addition of nutrients, electron acceptors, or other growth-stimulating factors to contaminated sites, thereby promoting metabolic activity of indigenous microorganisms (Cycoń et al., 2017). Unlike bioaugmentation, which introduces exogenous microbial strains, biostimulation relies on the native microbial community and is therefore more cost-effective and environmentally sustainable (Romantschuk et al., 2023). The effectiveness of biostimulation depends on factors such as soil composition, contaminant type, and microbial diversity, which require tailored strategies for different sites (Romantschuk et al., 2023). A key aspect of biostimulation is nutrient supplementation, particularly nitrogen and phosphorus, which are known to be the limiting factors for microbial growth and pesticide breakdown (Aldas-Vargas et al., 2021; Kuppan et al., 2024). Studies have shown that adding these nutrients accelerates the degradation of pesticides, like chlorpyrifos, by increasing microbial biomass and enzymatic activity (Li X. et al., 2020). Carbon sources, such as molasses or simple sugars, stimulate co-metabolic degradation pathways in which microbes consume carbon and simultaneously degrade pesticides (Rana et al., 2019; Ahmad et al., 2022; Leskovac and Petrović, 2023). Adequate oxygen availability is also critical, and techniques such as soil aeration or use of oxygen-releasing compounds (ORCs) enhance microbial activity in oxygen-limited environments (Yu et al., 2024). Organic amendments, such as livestock manure or compost, can accelerate the breakdown of persistent pesticides by supplying essential nutrients, improving soil structure, and enhancing microbial habitats (Romantschuk et al., 2023). In addition, combined approaches such as biostimulation integrated with plant–microbe systems have shown promising results. For example, rhizosphere exudates, such as carboxylic acids, can enhance microbial degradation of recalcitrant pesticides in amended soils (Boutahiri et al., 2024).

### 3.4 Composting

Composting has become an effective and sustainable method for bioremediating soils contaminated with pesticides and other organic pollutants (Figure 2). The process operates optimally at thermophilic temperatures between 54 °C and 65 °C, which enhances microbial activity, enzymatic reactions, and overall degradation efficiency (Karimi et al., 2022; Ataikiru and Ajuzieogu, 2023). Composting proceeds through four distinct stages: mesophilic, thermophilic, cooling, and maturation. During the mesophilic phase, microbial decomposition of organic matter generates heat, increasing the temperature to around 55 °C. The subsequent thermophilic stage is characterized by the dominance of heat-tolerant bacteria and actinomycetes that are capable of degrading complex organic compounds, such as lignin, cellulose, and residual xenobiotics (Wong and Wong, 2023). As nutrients become limited, temperatures gradually decline during the cooling phase, allowing mesophilic fungi and bacteria such as *Aspergillus, Penicillium, Bacillus*, and *Pseudomonas* species to continue decomposition, while maturation stage stabilizes the material into nutrient-rich, non-toxic compost (Zhang et al., 2022a; Aguilar-Paredes et al., 2023). The mechanisms of pesticide degradation during composting involve both adsorption and microbial transformation. Adsorption onto organic matter or added materials such as straw, manure, or wood chips reduces pesticide bioavailability, while microbial metabolism converts these compounds into simpler, less toxic products (Ataikiru and Ajuzieogu, 2023). Thus, composting offers a cost-effective and environmentally viable solution for detoxifying pesticide-contaminated soils, particularly when combined with biochar or nutrient-amended systems that enhance microbial resilience and enzymatic activity.

### 3.5 Mycoremediation

Mycoremediation uses fungi to degrade pesticide residues in contaminated environments through enzymatic and metabolic processes (Dinakarkumar et al., 2024). Fungal community succession studies indicate that species belonging to *Aspergillus* and *Penicillium* predominate during soil remediation and composting stages, where oxidative and hydrolytic enzymatic activities remain high (Swathy et al., 2024). For example, *Aspergillus sydowii* CBMAI 935, isolated from marine environments, acts as a biocatalyst for degrading chlorpyrifos and profenofos, highlighting its potential for bioremediation applications (González-Abradelo et al., 2019). Fungal species like *Bionectria antennata* reduce diazinon concentrations by 83.88% within 10 days, leveraging pesticides as carbon sources (Njoku et al., 2020). *Pleurotus ostreatus* and *Aspergillus niger* have been widely reported to efficiently remediate pesticide-contaminated soils while maintaining soil fertility and avoiding secondary pollution (Dinakarkumar et al., 2024; Swathy et al., 2024). Enzymes such as laccases, peroxidases, and cytochrome P450 monooxygenases play critical roles in fungal breakdown of organochlorine and organophosphate pesticides (Thirumalaivasan et al., 2024). These enzymes catalyze oxidation, dechlorination, and hydrolysis of toxic compounds, transforming them into less persistent and less toxic intermediates. The integration of fungal–bacterial consortia has been shown to further improve degradation rates by expanding the metabolic range and increasing resilience under variable soil conditions (Thirumalaivasan et al., 2024).

### 3.6 Anaerobic degradation

Anaerobic degradation of pesticides under oxygen-limited conditions involves metabolic activities of diverse microorganisms utilizing alternative electron acceptors, such as nitrate, sulphate, ferric iron, and carbon dioxide (Ghattas et al., 2017; Li et al., 2021). This degradation route is particularly important for persistent and recalcitrant pesticides that resist rapid aerobic breakdown. In nitrate- and sulphate-reducing environments, microbial communities can cause reductive dechlorination of organochlorine compounds like lindane and pentachlorophenol via sequential chlorine removal by specialist bacteria such as those belonging to the genus *Dehalococcoides* (Li et al., 2021). For example, organohalide-respiring bacteria express reductive dehalogenase enzymes that catalyse removal of halogen substituent under strict anaerobic conditions (Ghattas et al., 2017; Yan et al., 2024). Engineered anaerobic bioremediation systems, including substrate amendments, microbial bioaugmentation and permeable reactive barriers have demonstrated enhanced removal of chlorinated pesticide residues in soil and groundwater by creating low-redox zones conducive for dehalogenation (Li et al., 2021). Under anoxic conditions, microbial hydrolysis, mineralization, and co-metabolic pathways can facilitate the breakdown of organophosphate or carbamate pesticides also, although these routes are less well-characterized than those for halogenated compounds (Ghattas et al., 2017).

### 3.7 Land farming and biopiles

Land farming is an *ex-situ* bioremediation method in which contaminated soil is excavated, spread over a prepared bed, and periodically tilled to enhance aeration and microbial activity (Raffa and Chiampo, 2021). The method relies on indigenous microorganisms which degrade organic pollutants through oxidative and enzymatic processes in the presence of oxygen. Although it is low in cost and relatively simple to operate, the remediation period can be long, and the process poses risk of contaminant leaching or volatilization under uncontrolled conditions (Michael-Igolima et al., 2022). The efficiency of land farming depends on parameters, such as temperature, moisture, soil texture, pH, and nutrient balance. Nutrient and organic amendments, including compost, manure, and straw can significantly enhance microbial degradation activity by improving soil structure and nutrient availability (Lau, 2023). These co-amendments promote the proliferation of hydrocarbon- and pesticide-degrading bacteria, thereby increasing enzymatic activity involved in contaminant transformation.

Biopiles are more engineered and space-efficient alternative to land farming, combining controlled aeration, moisture regulation, and nutrient amendment to accelerate contaminant degradation (da Sales Silva et al., 2020). In biopile systems, contaminated soil is heaped on a lined platform, aerated either passively or through mechanical blowers, and supplemented with nutrients to sustain microbial metabolism (Alori et al., 2022) Figure 2. The addition of bulking agents, such as sawdust, compost, or biochar, improves porosity and oxygen transfer, thereby enhancing degradation of persistent pesticides, including DDT, endosulfan, and atrazine (Lau, 2023; Bala et al., 2022). Field-scale applications have demonstrated that integrating organic co-substrates or surfactant-modified soils into biopile systems can remove more than 80% of mixed pesticide residues within three months (Raffa and Chiampo, 2021; Mekonnen et al., 2024). The combination of microbial bioaugmentation further improves efficiency, making biopiles suitable for the remediation of time-sensitive and highly contaminated soils (Raffa and Chiampo, 2021). Compared with land farming, biopiles typically require less space and shorter treatment times. However, higher capital investment and continuous monitoring of temperature, aeration, and moisture is necessary to maintain optimal microbial performance.

### 3.8 Bioslurry reactors

Slurry bioreactors represent one of the most advanced *ex-situ* soil bioremediation methods, offering precise control over process variables such as pH, temperature, dissolved oxygen, mixing intensity, hydraulic retention time, and nutrient balance, which together govern microbial activity and contaminant bioavailability (Sun et al., 2023). In operation, contaminated soil is typically excavated, screened to remove oversized debris, and then mixed with water to form homogeneous slurry, which improves contact among microorganisms, nutrients, and pesticide molecules and reduce bioavailability constraints (Perez-Vazquez et al., 2024; Aljabri, 2025). Because mixing and phase transfer are strengthened in slurry systems, rate limitations linked to contaminant desorption and mass transfer are alleviated compared to less controlled soil treatments (Alori et al., 2022; Nie et al., 2024). These systems may operate under aerobic, anoxic or anaerobic conditions, and can run in batch, semi-continuous or continuous modes depending on the contaminant type and remediation goal. Slurry bioreactors have been used effectively for the treatment of herbicides, pesticides, explosives and polycyclic aromatic hydrocarbons, especially when supplemented with additional electron acceptors or carbon substrates to stimulate microbial communities (Sun et al., 2023). For example, recent work on bio-slurry reactors has demonstrated that soil-to-water ratios in the range of 1:10 to 1:20 along with the addition of co-substrates significantly enhanced the removal of dimethoate and similar organophosphates (Guowen et al., 2023). Although promising, high capital and operational costs of slurry bioreactor systems restrict their widespread application until medium- or full-scale field demonstrations become more common. Moreover, microbial community characterization within these systems remains under-explored. Additional constraints often include slurry handling logistics, energy requirements for mixing and aeration, management of spent water and fine solids, and need to scale laboratory-optimized conditions to variable field soils without loss of performance (Perez-Vazquez et al., 2024; Sun et al., 2023). In this context, greater use of microbial communities along with linking of key degraders having catabolic genes is important for improving predictability and designing robust operational strategies across pesticide classes (Raffa and Chiampo, 2021; Sun et al., 2023).

## 4 Biotechnological foundations of microbial consortia for pesticide degradation

The widespread use of chemical pesticides has led to significant adverse impacts on the environment, prompting the use of various physico-chemical and microbial techniques to eliminate pesticide contamination. However, compared to physico-chemical methods, microbial approaches have proven to be more robust and effective due to their efficiency and eco-friendly nature. From a microbiological perspective, many researchers have focused on axenic cultures for studying microbial pesticide degradation (Qian et al., 2019). Various gene expression pathways, metabolic pathways, and functional proteins have been identified in culturable microbes, which play a pivotal role in pesticide degradation. However, studies suggest that combined microbial applications exhibit a higher level of bioremediation efficiency compared to monoculture approaches (Lee et al., 2018; Qian et al., 2019; Bhatt et al., 2021; Zhang and Zhang, 2022). Traditional metabolic engineering using pure cultures provides essential insights into microbial pathways and their key metabolic products (Lopes et al., 2022). The absence of genes and enzymes in silent metabolic pathways, along with stringent culture requirements, significantly limits yield and productivity of metabolic products (Bhatia et al., 2018). Various commercially available pure-cultured microbes have been used on a large scale as bio-decontaminating and soil- conditioning agents. However, literature suggests that approximately 99% of environmentally friendly microbes cannot be cultivated in laboratories using traditional techniques (Egelkamp et al., 2019).

Researchers have suggested that microbial consortia from diverse environments holds strong potential for degrading toxic chemical pesticides compared to single microbes (Qian et al., 2019). Previously, microbiologists have employed various genetic engineering approaches to upregulate biomolecules; however, these efforts have only achieved limited success for commercialization in large-scale remediation (Rebello et al., 2021). One major limitation of using a single microbial strain for pesticide toxicity reduction is its metabolic burden. Due to limited resources, a single strain is often unable to perform multiple tasks simultaneously. Pesticide-induced toxicity causes cellular stress, prompting the host cell to consume increased amounts of energy in the form of NADH (nicotinamide adenine dinucleotide) and ATP (adenosine triphosphate) (He et al., 2017; Vermelho et al., 2024). Pesticide-degrading microbial strains synthesize these energy-rich molecules through universal metabolic pathways (He et al., 2017). The combined effects of metabolic burden and cellular stress lead to a significant decline in microbial biosynthesis, a phenomenon known as metabolic cliff (Vermelho et al., 2024). To address the limitations of single microbial strains, microbiologists have developed an alternative approach known as microbial consortium strategy (Nunes et al., 2024). In terms of metabolic pathways, single eukaryotic strain often perform better due to compartmentalization within the eukaryotic cell, which plays a crucial role in mitigating the effects of the metabolic cliff. In microbial consortia, various strains coordinate through a division of labour, with each member assigned a distinct metabolic role. Some members are responsible for pesticide bioremediation, while others play a pivotal role in regulating the production of biochemical compounds within the cells (Ram et al., 2022; Nunes et al., 2024). Therefore, microbial consortia offer the most effective solution for pesticide degradation and managing metabolic load in contaminated environment (Li et al., 2022).

Various studies have demonstrated that microbial consortia can rapidly degrade pesticides in a better way compared to single microorganism (Roell et al., 2019) (Table 3). Mixed microbial strains have been shown to perform complex tasks more effectively than individual strains (Nunes et al., 2024). In pesticide degradation, these consortia enhance efficiency by sharing metabolic responsibilities through interconnected degradation pathways. Each strain within the consortium can independently carry out specific steps in the degradation process—tasks that may be too complex for a single strain to handle alone. In natural environments, microbial consortia exhibit greater resilience to environmental fluctuations compared to individual strains. They also tend to resist invasion by foreign microbial species more effectively (Harcombe et al., 2018). A crucial factor in the functionality of these consortia is intercellular communication, which defines the role of each strain during degradation. The primary mechanism for such coordination is quorum sensing (QS), wherein bacterial cells produce and respond to signalling molecules, primarily lactones that serve as diffusible signals. It has been reported that inoculation with a microbial consortium in different soil samples enhanced plant growth and pollutant remediation by approximately 48 and 80%, respectively, whereas inoculation with a single microbial strain resulted in only about 29 and 48% improvement in plant growth and pollutant remediation, respectively (Liu et al., 2023). As per the high rate of environmental remediation processes is concerned, microbial consortia is commonly used than the single strain. The microbial consortia have been reported to exhibit a higher degradation capacity for toxic compounds in soil and sewage compared to single microbial strain applications. Reports also suggest that consortia-treated soil significantly enhances the growth and vigor of various shrubs and trees, while consortia-treated sewage water can be safely utilized for irrigating non-edible commercial crops (Biswas et al., 2021). Microbial consortia possess a superior ability to degrade complex compounds such as starch and cellulose, which cannot be efficiently broken down by a single strain. Within the consortium, different microbial strains work synergistically—some degrade these complex polymers into simpler sugars, which then serve as carbon sources for other microorganisms in the group (Wang S. et al., 2019; Tondro et al., 2020).

**Table 3:** Role of microbial consortia in the degradation of pesticides under different environmental conditions.

Microbial consortium	Growth media	Pesticide degraded	Transitional compounds	References
Actinomycetes, Proteobacteria	Soil	Methyl parathion, Parathion	4-Nitrocatechol, 4-Nitrophenol	Paul et al. (2006)
*Pseudomonas* spp.	Minimal medium	Carbamate insecticide	1-Naphthol, catechol, salicylic acid	Chapalamadugu S and Chaudhry GR (1991)
Bacterial biofilm A, B, C, D	Minimal medium	Methyl Diclofop,	Diclofop, 4 (2,4dichlorophenoxy)-phenol	Wolfaardt et al. (1994)
*Serratia marcescens*	Minimal medium	Dichlorodiphenyltrichloroethane (DDT)	1,1-Dichloro 2,2 bis (4-chlorophenyl) ethane, 1,1,1-trichloro-2-o-chlorophenyl 2-pchlorophenylethane	Bidlan and Manonmani (2002)
Indigenous soil bacterial combination	Minimal medium, Soil	Carbendazim, 2,4 dichlorophenoxyacetic acid	2-Aminobenzimidazole,2- hydroxybenzimidazole	Pattanasupong et al. (2004)
Actinobacteria consortium	Soil with Lindane contamination	Lindane	1,24-Trichlorobenzene, 2,5-dichlorophenol,2,5- dichlorohydroquinone	Lin et al. (2020); Raimondo et al. (2020)
*Pseudomonas putida*, CFR1021, *P. fluorescens* CFR1022, *P. aeruginosa* CFR1023, *P. aeruginosa* CFR1024, *Burkholderia cepacia* CFR1025, *B. cepacia* CFR1026, *P. stutzeri* CFR1027, *Vibrio alginolyticus* CFR1028, *Acinetobacter lwoffii* CFR1029, *Fusarium* sp. CFR225	Minimal medium	Hexachlorocyclohexane	1,24-Trichlorobenzene, 2,5-dichlorophenol, 2,5- dichlorohydroquinone	Lin et al. (2020); Li et al. (2017)
Fungal consortium	Soil managed for rice cultivation	Chlorpyrifos (Organophosphate insecticide)	2-Hydroxy-3,5,6-trichloropyridine	Conde-Avila et al. (2021)
Equatic microbial communities	Morpholinepropanesulfonic acid synthetic medium	Glyphosate [N-(Phosphonomethyl)glycine]	Aminomethylphosphonic acid, acetylglyphosate, sarcosine	Artigas et al. (2020)
*Streptomyces* spp., strains AC5, AC9, GA11, ISP13	Minimal medium	Diazinon, chlorpyrifos (organophosphate insecticides)	3,5,6-Trichloro-2-pyridinol	Briceno G et al., 2016
*Arthrobacter sulfonivorans, Variovorax soli* and *Advenella* sp. JRO	Minimal medium	Diuron (Pre-emergent herbicide)	3,4-Dichlorophenylamine	Villaverde et al. (2017)
*Burkholderia*, *Sphingopyxis*, and *Variovorax* genera of bacteria	Soil & Minimal medium	2,4- Dichlorophenoxyacetic acid (synthetic herbicide); 4-nitrophenol (fungicide)	Ammonia, nitrite	Wang et al. (2017)
*Methylobacterium radiotolerans, Microbacterium arthrosphaerae*	Agricultural soil	Imidacloprid (Insecticide)	5-Hydroxymetabolit, 6-chloronicotinic acid, 6- hydroxynicotinic acid	Erguven and Yildirim (2019)
*Coriolus versicolor* NBRC9791, activated sludge mixed bacterial cultures	Nutrient medium	Aldicarb, atrazine, alachlor	Aldicarb sulfone, Hydroxyatrazine, cyanuric acid, deisopropylatrazine, 2- Chloro 2,6- diethylacetanilide	Dehghani et al. (2013); Bhatt et al. (2019); Hai et al. (2012)
*Streptomyces* sp., M7, MC1, A5 and *Mycolatopsis tucumanensis* DSM 45259	Soil & Minimal medium	Lindane (used to treat scabies and lice infestations)	1,2,4-Trichlorobenzene, 2,5-dichlorophenol, 2,5- dichlorohydroquinone	Aparicio et al. (2018); Zhang et al. (2020)
*Pseudomonas, Klebsiella, Stenotrophomonas, Ochrobactrum, Bacillus*	M9 medium, soil slurry	Chlorpyrifos (organophosphate insecticide)	3,5,6-Trichloropyridinol, diethylthiophosphate	Singh P et al. (2016)
*Ochrobactrum anthropi, Acinetobacter johnsonii, Pseudomonas* sp. and *Stenotrophomonas maltophilia*	Mineral salt medium	Clothianidin (neonicotinoid insecticide)	5-Amino-methlthiazol, N-(1,2-thiazole-5- ylmethyl)-N-methylguanidine, N-(2-chloro-1- 3 thiazole-5ylmethyl)-N-methylurea	Wang X. et al. (2019)
*Pseudoxanthomonas indica, Bacillus anthracis, Bacillus cereus*	Mineral medium, soil slurry	Diuron (Pre-emergent herbicide)	3,4-Dichloroaniline	Villaverde et al. (2018)

Książek-Trela et al. (2025) reported a high degradation rate of herbicide diflufenican (DFF) (approximately 70.1%) when a single strain, *Streptomyces atratus* (strain ROA017-D1), was inoculated in a liquid medium. However, in the same medium, supplementation of a synthetic bacterial consortium consisting of four strains—*Pseudomonas* sp. 10Kp8 (A1), *Pseudomonas chlororaphis* subsp. *aureofaciens* strain B19 (A2), *Pseudomonas baetica* strain JZY4-9 (C1), and *Streptomyces atratus* strain ROA017 (D1)—resulted in a higher degradation rate of DFF, reaching about 74.4%. Furthermore, the application of strain D1 alone in soil medium achieved approximately 79% degradation of DFF, whereas the consortium of four strains exhibited the highest degradation efficiency, reaching around 82.2%. The researchers explained that consortium-based biodegradation of DFF proceeded through three sequential steps: (a) Step I (Initiation Phase) was mediated by *Pseudomonas* sp. 10Kp8 (A1) and *Pseudomonas chlororaphis* subsp. *aureofaciens* B19 (A2). These metabolically versatile strains employed monooxygenases, dioxygenases, and amidases to initiate DFF transformation through aromatic ring hydroxylation and amide bond cleavage, generating less toxic, more polar intermediates that were accessible to downstream degraders; (b) In Step II (Defluorination and Intermediate Transformation), *Pseudomonas baetica* strain JZY4-9 (C1) further metabolized these intermediates. This strain facilitated reductive or co-metabolic defluorination and additional ring-cleavage reactions, converting fluorinated pyridine derivatives into simpler, low-molecular-weight organic acids and preventing the accumulation of persistent metabolites; (c) The final phase, Step III (Mineralization), was carried out by *Streptomyces atratus* strain ROA017 (D1). This actinomycete produced extracellular oxidative and hydrolytic enzymes, including laccases and peroxidases, which enabled complete breakdown of residual aromatic structures and mineralization into CO₂, H₂O, NH₄^+^, and simple organic acids (Książek-Trela et al., 2025). Overall, the enhanced degradation of DFF by this four-member consortium resulted from sequential and complementary metabolic interactions, wherein *Pseudomonas* strains initiated transformation, *P. baetica* detoxified fluorinated intermediates, and *S. atratus* completed mineralization. This cooperative mechanism underscores the ecological and biotechnological potential of microbial consortia for bioremediation of persistent fluorinated herbicides.

Communication within the consortium often occurs through biofilm formation, enabling efficient cell-to-cell signalling—an essential feature for the development of synthetic microbial consortia. This intercellular communication system is modular and engineerable, offering a foundation for the rational design of synthetic consortia. However, consortia are not always beneficial; in some cases, they may produce inhibitory compounds that are toxic to the member strains, impeding their growth and functionality. Therefore, careful design and selection of strains is critical for developing effective and stable microbial consortia for pesticide degradation.

## 5 Genetics and genetic engineering of microbes for enhanced pesticide degradation

**Figure 3:**
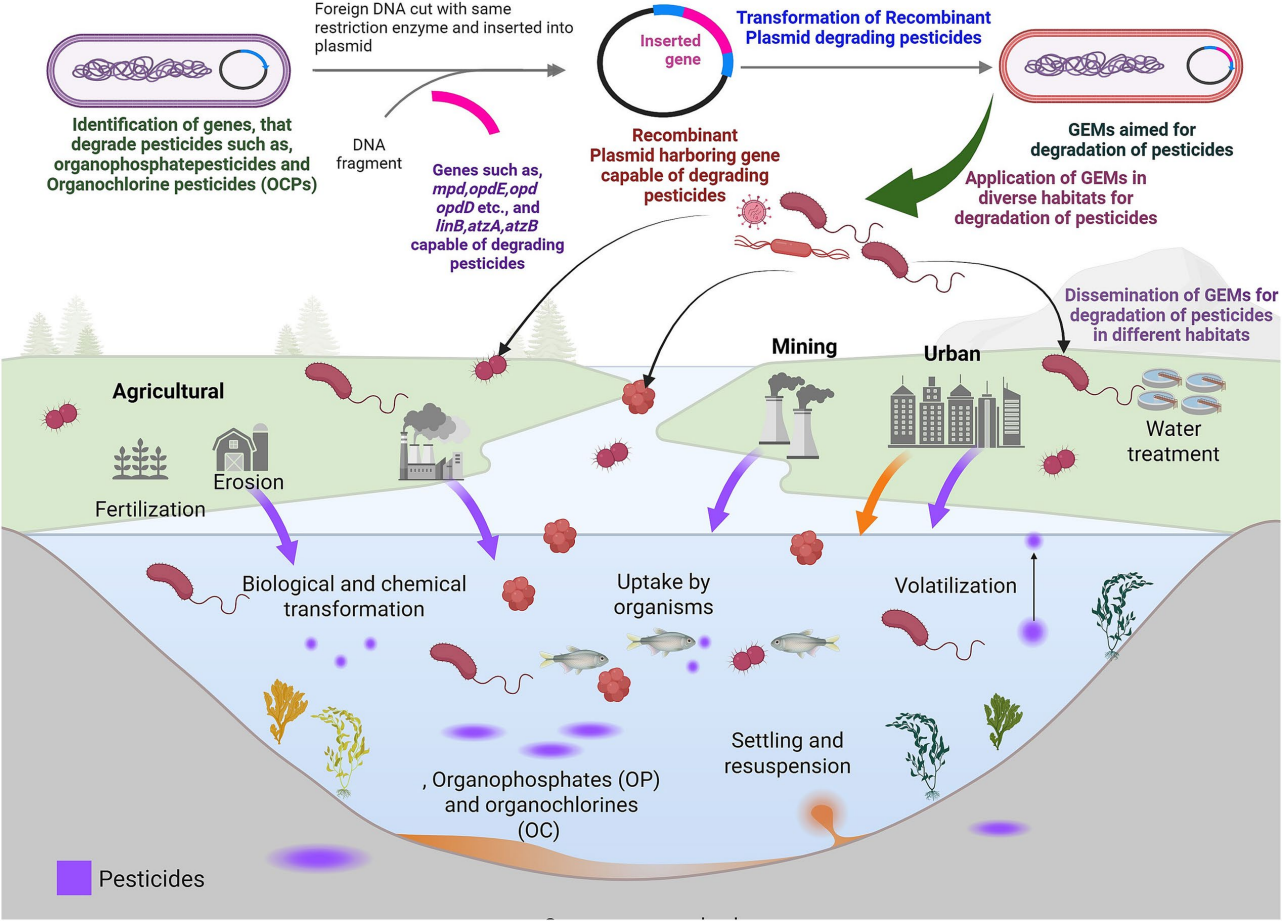
Microbial genetic engineering strategies for degradation of organochlorine pollutants: figure depicts dissemination of organochlorine pesticides (OCPs) and organochlorines (OC) as persistent pollutants toxic to biological systems. GEMs are developed to increase the production of genes and their products aiming to degrade a specific type of pesticide. This approach alters the microbial strains to breakdown the pollutants which is an effective solution to ineffective decomposition of pollutants by traditional approaches.

Advancements in recent technologies for the degradation of pesticides have led to the clean-up of various habitats due to the sheer limitations in conventional procedures. Microbes play pivotal roles in growth and development of plants, animals and other microbes (Góngora-Echeverría et al., 2020; Bhatt et al., 2021; Ali et al., 2022; Gul et al., 2023; Tyagi et al., 2024). Hence, using and manipulating the genetic information of microbes plays a pivotal role in mitigating the eco-toxicity mediated by pesticides, which in turn leads to various diseases in humans. Recent approaches have focussed on the production of genetically engineered microorganisms (GEMs) to increase the production of genes and their products with an aim to degrade a specific type of pesticide (Rafeeq et al., 2023) (Figure 3). The alteration of microbial strains for the breakdown of the pollutants is believed to be an effective solution for the ineffective decomposition of pollutants by traditional approaches (Mishra et al., 2020; Peng et al., 2020).

Pesticides such as organophosphates (OP) and organochlorines (OC) are persistent hazards that have significant negative impacts on human health and also destabilize the environmental homeostasis. For instance, the OPs act as potent neurotoxins by inhibiting the enzyme acetylcholine esterase (Singh and Verma, 2024). Additionally, the Hexachlorocyclohexane (HCH) is reported to accumulate and severely impair the functioning of kidneys, nervous system and liver (Yadav and Kumar, 2024). A wide range of research supports the existence of genes critical for catabolizing OPs from various habitats (Table 4). The bacterial enzymes are the major players in detoxifying pesticides effectively and at low costs. For example, ethyl parathion degrading enzyme (*mpd*) encoded by *Plesiomonas* sp. strain genome is able to hydrolyse a wide range of thion and oxon OPs (Khan et al., 2025; Mahmoud A. M. et al., 2025; Mahmoud G. A. E. et al., 2025). Methyl parathion hydrolase (MPH) enzyme targets the central phosphorus atom of organophosphates and break phosphoester bond in specific P-O or P-S aryl bond (Dong et al., 2005). Upon binding to OPs, such as ethyl parathion or methyl parathion, the phosphate group is removed by using a water molecule in active site through nucleophilic attack. This leads to hydrolytic cleavage of P–O bond (the aryl ester bond) leading to the formation of less toxic by products such as, 4-Nitrophenol (p-nitrophenol) and diethyl thiophosphate from ethyl parathion or dimethyl thiophosphate from methyl parathion (Mahmoud A. M. et al., 2025; Mahmoud G. A. E. et al., 2025). Additionally, the *mpd* produced by bacteria can use pesticides as their nutritive source. Researchers have engineered *Sphingomonas* sp. with gene encoding *mpd* (methyl parathion hydrolase) derived from *Pseudomonas putida* to degrade OP and carbamate pesticides in a wide range of habitats (Xu et al., 2020). Similarly, the gene *pytH* encoding a non-specific carboxylase esterase was cloned from *Sphingobium* sp. strain JZ-1 to degrade wide variety of pyrethroids (Xu et al., 2020). This enzyme with a molecular weight of 31kDA, belonging to alpha/beta-hydrolase (ABH) fold superfamily, consists of catalytic triad of Ser78, His230, and Asp202 in its active side (Xu et al., 2020). Further, the hydrophobic pocket in *pytH* enzyme which is large and deep consisting of 29 amino acid residues, allows this enzyme to accommodate complex and bulky pesticides such as bifenthrin and permethrins for degradation (Xu et al., 2020). The His230 residue in catalytic pocket of *pytH* deprotonates hydroxyl group of Ser78, which in turn performs nucleophilic attack on carbonyl carbon of the pyrethroid’s ester bond (Xu et al., 2020). The resulting tetrahedral transition state is further stabilized by “oxyanion hole,” consisting of nitrogen atoms as backbone. Later mechanism leads to the release of alcohol moity (3-phenoxybenzyl alcohol) from pesticide, whereas the acid moity remains covalently attached to the enzyme. Further, the deprotonated His230 becomes hydroxide ion, which in turn attacks the acyl-enzyme intermediate to create a second tetrahedral intermediate. Subsequently, the intermediate collapses and releases the acid moity to restore the native state of *pytH* enzyme (Xu et al., 2020). The *pytH* enzyme has wide range of effectiveness against various pyrethroids such as, deltamethrin, permethrin and cypermethrin. In addition, *pytH* does not show specificities to chiral isomers, evolving it as a robust tool for complete detoxification of pesticides (Zhan et al., 2020).

**Table 4:** Genes and their products released by microorganisms to degrade the organophosphate pesticides and organochlorine pesticides (OCPs) in soil.

Microorganism	Gene	Encoding enzyme	Target pesticide	References
Organophosphate pesticides (OPs)				
*Plesiomonas* sp. M6	*mpd*	Methyl parathion hydrolase (MPH)	Chlorpyrifos	Cui et al. (2001)
*Enterobacter* sp.	*opdE*	Phosphotriesterase hydrolase	Methyl parathion, Phorate, Parathion	Shen et al. (2010)
*Agrobacterium radiobacter* P230	*opd*	Phosphodiestrase	Phosmet and Fenthion	Horne et al. (2002)
*Lactobacillus sakei* WCP904	*opdD*	Organophosphorus hydrolase	Chlorpyrifos	Gao et al. (2012)
*Ochrobactrum* sp. JAS2	*mpd*	Methyl parathion hydrolase (MPH)	Chlorpyrifos	Cui et al. (2001)
*Pseudomonas aeruginosa*	*phd*	PhdA (prevent-host-death family protein)	Parathion	Mulbry and Karns (1989)
*Cupriavidus* sp. DT-1	*mpd*	Methyl parathion hydrolase (MPH)	Chlorpyrifos	Lu et al. (2013)
*Stenotrophomonas* sp. SMSP-1	*ophc2*	Organophosphorus hydrolase	Methyl parathion	Horne et al. (2002)
*Lactobacillus brevis* WCP902	*opdB*	Organophosphorus hydrolase (OpdB)	Chlorpyrifos, Coumaphos, Diazinon, Methyl parathion, and Parathion	Wang et al. (2018)
*Ochrobactrum* sp., *Pseudomonas putida* KT2440	*tpd*	Triazophos hydrolase	Triazophos, Methyl parathion	Islam et al. (2010) and Zhong and Chen (2006), Cui et al. (2001)
*Serratia marcescens* MEW06	*mph*	Methyl parathion hydrolase (MPH)	Dimethoate, Paraoxon, Methyl parathion	Chino-Flores et al. (2012)
*Pseudomonas* sp. strain ADP	*atzA*	Atrazine chlorohydrolase	Atrazine	Neumann et al. (2004)
*Sphingobium quisquiliarum* DC-2	*cmeH*	Amidase	Acetochlor	Li et al. (2013)
*Sphingobium* sp. JZ-2	*pytH*	Pyrethroid hydrolase	Fenpropathrin	Duan et al. (2011)
*Rhodococcus* sp. T1	*feh*	Fenoxaprop-ethyl hydrolase	Fenoxaprop-ethyl	Hou et al. (2011)
*Ochrobactrum* sp.mp-4	*tpd*	Triazophos hydrolase	Triazophos concentration	Gu et al. (2006)
*Plesiomonas* sp. strain M6	*mpd*	Methyl parathion hydrolase	Methyl parathion	Zhongli et al. (2001)
*Pseudomonas pseudomallei*	*glpA/glpB*	Glycerol-3-phosphate dehydrogenase	Glyphosate	Peñaloza-Vazquez et al. (1995)
Organochlorine pesticides (OCPs)				
*Sphingobium japonicum* UT26	*linB*	Haloalkane dehalogenase,	Haloalkanes and related compounds	Nagata et al. (1997)
*Pseudomonas* sp. ADP	*atzA*	Atrazine chlorohydrolase (AtzA)	Atrazine	de Souza et al. (1996)
*Pseudomonas* sp. ADP	*atzB*	Hydroxyatrazine ethylaminohydrolase	2-chloro-4-amino-6- hydroxy-*s*-triazine	Seffernick et al. (2002)
*Arthrobacter aurescens* TC1	*trzN*	Amidohydrolase	Atrazine	Mulbry et al. (2002)
*Sphingobium chlorophenolicum* L-1	*pcpC*	TCHQ-reductive dehalogenase	TeCHQ, 2,3,6-TCHQ	Orser et al. (1993)
*Sphingobium japonicum* UT26	*linD*	Reductive dechlorinase	2,5-DCHQ, CHQ	Miyauchi et al. (1998) and Nagata et al. (2016)
*Sphingobium japonicum UT26*	*linA*	Dehydrochlorinase	HCHs and its metabolites	Nagata et al. (1993) Nagata et al. (2016)
*Sphingobium japonicum* UT26	*linF*	maleylacetate reductase	2-CMA	Endo et al. (2005) and Nagata et al. (2016)
*Sphingobium chlorophenolicum* L-1	*pcpB*	Pentachlorophenol 4-monooxygenase	PCP	Orser et al. (1993) and Nagata et al. (2016)
*Sphingobium japonicum* UT26	*linE*	Hydrolase	2,6-DCHQ, CHQ	Miyauchi et al. (1999) and Nagata et al. (2016)

Genes coding for carbofuran hydrolase (*mcd*) and carbaryl hydrolase (*cehA*) were also cloned from *Achromobacter* sp. strain WM111 and *Rhizobium* sp. strain AC100, respectively, for pesticide degradation (Zhu et al., 2018). Previously, researchers identified oxon and thion organophosphates (OPs) degrading enzymes encoded by *Plesiomonas* sp. strain. Also the OCs degrading gene *linA* coding enzyme *γ*-hexachlorocyclohexane dehydrochlorinase from *Sphingomonas paucimobilis* UT26 has been reported to catalyse the γ-hexachlorocyclohexane (γ-HCH) to 1,2,4-trichloro benzene (1,2,4-TCB) via γ-1,3,4,5,6-pentachlorocyclo hexene (γ-PCCH) (Deng et al., 2024). The construction of a recombinant *E. coli* strain overexpressing the enzyme methyl parathion hydrolase (*MPH*) was able to efficiently degrade methyl parathion (Xu et al., 2022). Yang et al. (2012) constructed the genetically engineered *E. coli* strain expressing both *MPH* and *LinA* aimed to degrade the OPs and OCs with ease and efficiency. The genetically modified *E. coli* constructed to express fusion protein of Enhanced Green Fluorescent Protein (EGFP) and an Organophosphate Hydrolase (OPH), exhibited strong hydrolase activity to degrade the organophosphorus pesticide in the environment (Lourthuraj et al., 2022). In conclusion, nature has endowed us with huge number of microbiomes that could be manipulated to eradicate the toxic metabolites from the environment. These GEMs can play pivotal role in decreasing the concentration of pesticides in food webs.

Organochlorine pesticides (OCPs) are also persistent pollutants that have drawn much attention due to their toxic and persistent biological effects (Liu et al., 2025; de Souza Pomacena et al., 2025; Wang et al., 2024). Among all the existing organochlorine pesticides, the aldrin, *α*-, *β*-, and *γ*-hexachlorocyclohexane (HCH), 1,1,1-trichloro-2,2-bis(4-chlorophenyl) ethane (DDT), chlordecone, chlordane, dieldrin, endrin, heptachlor, hexachlorobenzene (HCB), mirex, pentachlorobenzene, and toxaphene are the most dominantly affecting the living systems (Tzanetou and Karasali, 2022). These OCPs have been proven to be hormone disruptors in humans. Several studies have been carried out to devise strategies for degrading the OCPs in environment. Further, a large number of genes have been reported to degrade a wide range of OCPs (Table 4). For instance, the gene *linA* coding for dehydrochlorinase derived from *Pseudomonas paucimobilis* UT26 was cloned into *Escherichia coli* to mediate the degradation of γ-HCH to 1,3,5, or 1,2,4-trichlorobenzene via tetrachorohexadiene (TCDN) and γ-pentachlorocyclohexane (γ-PCCH) (Yusuf et al., 2023: Deng et al., 2024). *LinA* (member of NTF2-like superfamily) mediates its enzymatic activity through stereochemical arrangement of its substrates. This enzyme requires 1, 2-trans-diaxial arrangement coordinated between hydrogen and chlorine atoms on the cyclohexane ring (Trantírek et al., 2001). The active site of *LinA* consists of catalytic dyad formed by Asp25 and His73, whereas His73 acts as a catalytic base for the removal of protons from the pesticide substrate (Manna et al., 2015). During this E2 elimination mechanism, the chloride ion is removed from the opposite rings to form carbon–carbon double bond. The *LinA* catalyses the initial dehydrochlorination of γ-hexachlorocyclohexane (γ-HCH) to form γ-pentachlorocyclohexene (γ-PCCH). Consequently, three axial chlorine substituents in γ-HCH, two distinct 1,2-diaxial H–Cl pairs are available for elimination to aid catalysis. Further, the *LinA* exhibits stringent stereochemical control by selectively targeting one of these pairs, resulting in the formation of a single, well-defined enantiomer of γ-PCCH. In a subsequent reaction, *LinA* catalyses a second dehydrochlorination, and removes an additional HCl moiety from γ-PCCH to produce 1,3,4,6-tetrachloro-1,4-cyclohexadiene (1,4-TCDN). The outcome of this step is to increase the degree of unsaturation of the cyclohexane ring through incorporation of a second double bond. The resulting metabolite, 1,2,4-trichlorobenzene (1,2,4-TCB), is converted to less toxic chlorinated catechols (like 3,4,6-trichlorocatechol) by enzymes chlorobenzene dioxygenase/monooxygenase, which introduces hydroxyl groups to 1,2,4-TCB ring. Subsequently, the dihydrodiol dehydrogenase rearomatizes the dihydrodiol intermediate. The later product is further degraded by chlorocatechol-1,2-dioxygenase (TcbC) to catechol ring (Brahushi et al., 2017).

Previous studies revealed that genes *LinA1* and *LinA2* encoding two variants of hexachlorocyclohexane dehydrochlorinase are responsible for degradation of lindane (Heeb et al., 2021; Deng et al., 2024). In another study, *Cupriavidus necator* JMP134 was reported to degrade 2,4-D by stepwise conversion from 2,4-dichlorophenol (2,4-DCP) by 2,4-D dioxygenase encoded by *TfdA* gene, and 2,4-DCP was further degraded to β-ketoadipate by 2,4-DCP hydroxylase encoded by *TfdB*, chlorocatechol dioxygenase encoded by *TfdC*, chloromuconate cycloisomerase encoded by *TfdD*, dienelactone hydrolase encoded by *TfdE*, and chloromaleylacetate reductase encoded by *TfdF* gene. The end-product β-Ketoadipate was shuttled to tricarboxylic acid (TCA) cycle for further metabolism (Pettinato et al., 2022). The hydrolases encoded by *atzABCDEF*, located on 108-kb IncP-1 β plasmid pADP-1 of *Pseudomonas* sp. strain ADP was reported to degrade the atrazine in six steps (Medić and Karadžić, 2022). Different groups of researchers have engineered *Pseudomonas putida* KT2440 to mineralize pesticides such as 1,2-dichloroethane (DCA), 1,2,3-trichloropropane (TCP), γ-hexachlorocyclohexane (γ-HCH), p-nitrophenol (PNP) and methyl parathion (MP) (Gong et al., 2016; Zhao et al., 2021; Huo et al., 2022, 2023). Wang et al. (2024) produced a novel *E. coli* strain BL-3164 by reconstructing pET-3164 plasmid containing a complete set of genes responsible for degradation of 2, 4-D. Recently, Xiong et al. (2025) engineered *Halomonas cupida* for efficient mineralization of 2,4-D in highly saline waste waters. These studies clearly indicate the repository of genes from natural sources that could be exploited to regulate the levels of pesticides in natural habitats.

The heterologous expression of carboxylesterase B1 (*CarE B1*) gene, derived from mosquito *Culex pipiens quinquefasciatus* was employed to degrade pesticides such as, pyrethroids and organochlorines (Li Q. et al., 2020). The *CarE B1* enzyme catalyses the hydrolysis of ester bonds found in pesticides. For instance, the cleavage of ester linkages in organophosphates and carbamates neutralizes their neurotoxic potential. Similarly, *CarE B1* enzyme breaks pyrethroids and organochlorines into less toxic polar metabolites. The neurotoxic organophosphates such as, chlorpyrifos, parathion can be degraded by employing GEMs such as *Escherichia coli* and *Pseudomonas putida* overexpressing *opd* gene derived from *Flavobacterium* or *Pseudomonas*. The *opd* genes targeted to periplasm or cell surface of GEMs encodes organophosphorus hydrolase (OPH) which cleaves phosphotriester bond in pesticides for detoxification (Li Q. et al., 2020). GEMs, such as, *Bacillus subtilis* have been genetically modified to express *gat* gene encoding glyphosate N-acetyltransferase and *gox* encoding glyphosate oxidoreductase. The glyphosate N-acetyltransferase catalyses the transfer of acetyl groups to pesticides making them less toxic to plant and animal systems. Similarly, the *gox* gene product glyphosate oxidoreductase cleaves C-N bonds in pesticides to produce glyoxylate and aminomethylphosphonic acid (AMPA).

The pyrethriods and carbamamte pesticides are degraded by GEMs *Bacillus megaterium* and *Pichia pastoris* (yeast) overexpressing *mpd* derived from insects. The *mpd* enzyme hydrolyzes the ester linkage resulting in reduction of half life of pesticides such as, cypermethrin and fenpropathrin (Tutika and Himabindu, 2025). Overall, the construction of GEMs plays a pivotal role in clearing pesticides from the environment. The emerging techniques, such as antioxidant systems, use of microbial consortia and genome editing techniques such as CRIPSR/Cas systems, could be utilized to engineer the microbial genomes to mitigate the ecotoxicity induced by pesticides in a wide range of habitats (Barooah and Hazarika, 2022).

## 6 Benefits of microbial engineering for environment and economy

Pesticide contamination in soil is a significant environmental and agricultural challenge, leading to long-term ecological damage, reduced soil fertility, and risks to human and animal health (Zhou et al., 2025). Traditional methods for pesticide remediation, such as soil excavation, chemical treatments, or thermal desorption, are often expensive, inefficient, and environmentally disruptive (Liu M. et al., 2024). In contrast, microbial engineering offers a promising, sustainable, and cost-effective solution for cleaning pesticide residues in soil (Pant et al., 2021). Microbial engineering involves modifying microorganisms, often bacteria, actinomycetes, and fungi, through genetic or synthetic biological techniques to enhance their natural capacity to degrade toxic compounds. One of its primary advantages is the targeted degradation of persistent organic pollutants (POPs) such as organophosphates, carbamates, and chlorinated pesticides. Engineered microbes can break down these compounds into harmless substances, significantly reducing environmental toxicity (Kumar et al., 2025; Sadiq et al., 2025). Liu et al. (2016) developed a genetically modified strain of *Pseudomonas putida* capable of degrading chlorpyrifos, a widely used organophosphate pesticide. The engineered strain showed very good degradation efficiency, outperforming native microbial populations. Similarly, recently, *Bacillus subtilis* strains engineered to express organophosphorus hydrolase (OPH) have shown enhanced capability to detoxify various pesticides (Bahrulolum and Ahmadian, 2025). Li Q. et al. (2020) constructed a genetically engineered bacterium having the ability to degrade pesticides like organochloride, organophosphorus, carbamates, and pyrethoid insecticides. Another advantage of microbial engineering is its role in restoring soil health and biodiversity. Unlike harsh physical or chemical remediation techniques, bioengineered microbes can detoxify pollutants while maintaining or even improving soil structure and microbial diversity. This helps preserve beneficial soil functions, including nutrient cycling and plant-microbe interactions, which are essential for sustainable agriculture (Rebello et al., 2021; Karnwal et al., 2025). Engineered microbes can also be designed for enhanced survival and activity in harsh soil conditions, such as extreme pH or temperature, where natural strains may fail. Through synthetic biology, traits such as stress resistance, biofilm formation, or root colonization can be introduced to improve microbial persistence and effectiveness in contaminated fields (Sudheer et al., 2020; Misu et al., 2025). Additionally, microbial engineering enables precision biodegradation, where specific enzymes are optimized or overexpressed to target particular pesticide molecules. This selective degradation reduces the risk of non-specific microbial activity that might otherwise affect non-target compounds or disrupts soil ecosystems (Bittencourt et al., 2023; Qattan, 2025).

The economic viability of using engineered microbes is another key advantage. Compared to mechanical or chemical methods, microbial solutions are relatively low-cost, scalable, and require minimal labour or infrastructure. Once established, these microbes can continue to function over extended periods, providing long-term soil decontamination (Wend et al., 2024; Luan et al., 2025). In agriculture, engineered microbes are revolutionizing crop production. For instance, genetically modified rhizobacteria can enhance nitrogen fixation in non-leguminous crops like wheat and maize. This innovation reduces the dependency on synthetic nitrogen fertilizers, which are both costly and damaging to environment (Mayung, 2024). According to Kumar S. et al. (2022), deploying engineered *Azospirillum* strains in Indian wheat fields reduced fertilizer usage by up to 40%, saving farmers an estimated $75 per hectare. Similarly, engineered phosphate-solubilizing bacteria are improving nutrient availability, cutting back on the use of expensive phosphate fertilizers (Guo et al., 2023). Another economic advantage lies in pest and disease control. Engineered microbes such as *Bacillus thuringiensis* (Bt) and other biocontrol agents can be tailored for targeted pathogen suppression, reducing the need for chemical pesticides. This not only lowers input costs but also boosts crop quality and export potential. A study by Abbey et al. (2021) demonstrated that using a microbial bio-fungicide in blueberry farming resulted in a 20% yield increase and a significant reduction in fungicide expenditures.

Despite these benefits, regulatory hurdles, biosafety concerns, and public perception of GEMs remain a big challenge. However, new developments in bio-containment strategies, such as genetic kill-switches or dependency on synthetic nutrients, are helping to address these issues and improve environmental safety (Lea-Smith et al., 2025). Finally, it can be concluded that microbial engineering offers a highly effective and environment friendly approach for cleaning pesticide-contaminated soils. Its ability to degrade persistent pesticides, restore soil quality, and offer long-term, cost-effective remediation makes it a vital tool in the movement toward sustainable agriculture and environmental health.

## 7 Innovative technologies and patentable advances

Recent advances in microbial biotechnology and genetic engineering offer transformative opportunities for sustainable pesticide remediation in agricultural soils. Several innovations emerging from this field can be translated into patentable technologies and scalable applications.

### 7.1 Synthetic microbial consortia for broad-spectrum degradation

A novel innovation involves designing a synthetic microbial consortia wherein different microbes perform complementary degradation steps. Such consortia can be engineered for metabolic cross-feeding, enhancing pesticide breakdown efficiency under variable soil conditions. A microbial consortium consisting of *Azospirillum*, *Cloacibacterium*, and *Ochrobacterium* completely degraded 50 mg L^−1^ glyphosate within 36 h in both sterilized and non-sterilized water–sediment systems (Zhang et al., 2024b). In a comparable study, a four-strain *Bacillus* consortium *B. amyloliquefaciens* CP28, *B. pumilus* CP30, *B. marisflavi* CP31, and *B. subtilis* CP34, achieved 91% degradation of 100 ppm chlorpyrifos after 6 days of incubation at 30 °C and pH 7, demonstrating its effectiveness in remediating chlorpyrifos-contaminated environments (Varghese et al., 2021). Patent claims may include optimised microbial compositions, ratios, and formulations that exhibit superior stability, resilience, and degradation kinetics compared to single strains.

### 7.2 Enzyme-based bio-formulations and immobilised systems

Purified or immobilised pesticide-degrading enzymes can be incorporated into bio-formulations for targeted soil application. Immobilization on nanoparticles, biochar, or biodegradable polymers enhances enzyme stability and reusability. Among the various remediation approaches, enzymatic degradation is considered a principal strategy for pesticide removal because it minimizes the formation of undesirable by-products (Jaiswal et al., 2025). For instance, immobilization of laccase on iron magnetic nanoparticles significantly enhanced the enzymatic conversion of chlorpyrifos, with optimal activity observed at 55 °C and pH 7 (Srinivasan et al., 2020). Similarly, organophosphorus acid anhydrolase-FL immobilized within alginate beads was employed to assess the degradation of ethyl paraoxon in organophosphate-contaminated water. The immobilized enzyme system outperformed the free enzyme; exhibiting substantially higher stability and a deactivation constant approximately fourfold lower than that of the soluble enzyme (Jaiswal et al., 2025). Patentable innovations include slow-release enzyme carriers, enzyme–nanocomposites, and smart delivery systems activated by soil pH or moisture.

### 7.3 Smart bioremediation platforms using biosensors

The integration of microbial biosensors with bioremediation systems represents a novel technological advancement. Engineered microbes can be designed to detect pesticide residues and simultaneously activate degradation pathways. Biosensors employ biological components such as enzymes, nucleic acids, or whole cells for pollutant detection, with each biosensor type offering distinct advantages in terms of sensitivity, specificity, and response time. Amperometric enzyme-based biosensors have been successfully applied for the detection of polybrominated diphenyl ethers (PBDEs), achieving a limit of detection (LOD) as low as 0.014 μg L^−1^ in landfill leachate samples. In particular, glucose oxidase immobilized on gold nanoparticles enabled PBDE detection through enzyme inhibition, with an LOD of 0.14 μM. Whole-cell and microbial biosensors are widely used for heavy metal monitoring, exhibiting detection limits ranging from 0.1 to 1 μM (Sarti et al., 2025). Advances in synthetic biology have further enabled the development of engineered bacteria and fungi that function both as biosensing platforms and as agents for pollutant degradation (Zhu et al., 2023). *Pseudomonas* spp. have demonstrated effectiveness in detecting and degrading organic contaminants such as fluorene, phenanthrene, and benzene, achieving detection of 50 mg L^−1^ organic matter within 4 days and a degradation efficiency of 91.16% (Sankhyan et al., 2025). Additionally, enzyme-based biosensors are capable of detecting dyes at nanomolar concentrations and facilitate the oxidative degradation of phenolic compounds through catalytic activity (Nath, 2024). Patents may cover biosensor–bioremediator hybrid systems capable of real-time monitoring and self-regulated pesticide detoxification (Thanigaivel et al., 2025).

### 7.4 Rhizosphere targeted bioremediation tools

Innovations targeting the rhizosphere offer dual benefits of pesticide degradation and plant growth promotion. Three plant growth–promoting rhizobacterial (PGPR) strains, *Pseudomonas chlororaphis* (M4C4–5), *Pantoea allii* (M1C5–1), and *Mammaliicoccus sciuri* (M1C4–15), have been reported to tolerate chlorpyrifos and malathion concentrations of up to 2,000 mg L^−1^. These isolates demonstrated substantial pesticide degradation efficiency, achieving an 81.76% removal rate, while simultaneously promoting plant growth (Komicho and Tipayno, 2025). We have also reported that PGPRs (B*acillus* sp. SWP1 and *B. safensis* SWP5) can enhance nutrient uptake (by phosphate solubilization), reduce pesticide stress, and restore soil health (Kumar et al., 2021b). According to Inthama et al. (2021), *B. aryabhattai* MoB09 showed the highest paraquat degradation at 30 °C (pH 7) and also promoted the growth of *Vigna unguiculata.* Engineered plant growth promoting rhizobacteria with pesticide-degrading traits can be patented for its use as biofertilizer–bioremediator hybrids.

### 7.5 Genetically engineered super-degrader microbes

One promising innovation is the development of genetically engineered microbial strains with enhanced pesticide-degrading capabilities. By overexpressing key degradative enzymes such as hydrolases, oxygenases, laccases, and dehalogenases, microbes can be tailored to rapidly mineralise persistent pesticides. CRISPR-Cas–based genome editing can be employed to insert multi-enzyme degradation pathways into a single microbial host. Diverse approaches can be employed to integrate multiple degradation abilities into one engineered bacterium, such as protoplast fusion, horizontal gene transfer, and homologous recombination. The genomes of *Psathyrella candolleana* and *Pseudomonas putida* were recombined using the protoplast fusion technique to construct a super-degraded strain with the ability to degrade 78.98% of PCP in polluted water (Chen et al., 2013). Many researchers have constructed *E. coli* using a surface display system to display different functional enzymes to degrade various pesticides like paraoxon (Latifi et al., 2015), carbaryl (Liu et al., 2022), and *λ*-cyhalothrin (Ding et al., 2022). Patents could focus on engineered microbial strains capable of degrading multiple pesticide classes (organophosphates, carbamates, neonicotinoids) simultaneously.

### 7.6 Omics-guided microbial engineering platforms

The use of metagenomics, proteomics, and metabolomics to identify novel pesticide-degrading genes and pathways is another patentable frontier. Recent advances in multi-omics technologies have profoundly enhanced our understanding of microbial pesticide biodegradation. Genomic analyses have uncovered operons and previously uncharacterized catabolic genes associated with pesticide degradation pathways (Rodríguez et al., 2020), whereas transcriptomic studies elucidated the regulatory networks activated in response to pesticide-induced stress (Islam et al., 2024). Metabolomics enables the identification of degradation intermediates and end products, offering valuable insights into complete mineralization processes (Wang et al., 2025). Proteomic analyses further complement these approaches by revealing the active enzyme machinery involved during biodegradation. Collectively, integrated omics strategies provide an unprecedented framework for linking microbial gene function with ecological performance. Databases and AI-driven platforms that predict microbial degradation potential and guide strain engineering could form the basis of proprietary technologies (Dhakal et al., 2025).

These innovations highlight how microbial engineering, synthetic biology, and smart delivery systems can revolutionize pesticide bioremediation. Several such patents have been filed and granted in the last 5 years (Table 5). Patenting of such technologies will accelerate their adoption, promote sustainable agriculture, reduce environmental contamination, and deliver long-term economic and ecological benefits (Table 6).

**Table 5:** Important patents filed in the area of microbial pesticide degradation in the last 5 years.

Patent no.	Description	Countries	Patent type	References
US12091653B2	Strain of *Glutamicibacter,* originating from insects, capable of efficiently degrading bifenthrin	USA	Granted Utility Patent	Long et al. (2024)
WO2023044345A1	Microbial electrochemical system combining engineered microbes to degrade organophosphate pesticides (e.g., parathion/paraoxon) with biosensing.	PCT (Int) (Patent Cooperation Treaty -international)	International Application	Erina et al. (2023)
CN116396911B	Bacterial strain + microbial inoculum specifically for treating pesticide wastewater with associated application methods/devices.	China	Granted Patent	Tianming et al. (2023)
CN117244935A	Method of degrading residual pesticides in soil using targeted microbial agents with controlled irrigation and soil humidity.	China	Patent Application	Wengang and Haishen (2023)
WO2024238848A2	Systems & compositions for managing pesticide resistance, including minicell-based biological agents that reduce pesticide resistance and which encompass degradation pathways.	PCT (Intl)	International Application	Shakeel et al. (2024)
US20200123076A1	Agricultural microbial inoculant compositions and uses thereof	PCT (Intl)	International Application	Bobeck and Pearce (2023)
US11214597B2	Stable dry powder composition comprising biologically active microorganisms and/or bioactive materials and methods of making	PCT (Intl)	International Application	Harel et al. (2022)
404587	Novel microbial formulation for Endosulfan bioremediation using *Burkholderia* sp. (MTCC 25118) to degrade alpha/beta endosulfan in soil into non-toxic metabolites.	India	Granted Patent	Prakash and Basu (2022)
407022	Bio-pesticide compositions and formulation from (*Citrullus colocynthis*) for insect control. (Product name: Thar Jaivik 41 EC). This bio-pesticide is effective against *Helicoverpa armigera*, *Spodoptera litura*, white fly and aphid with safe to natural enemies.	India	Patent Application	Haldhar et al. (2022)

**Table 6:** Recommendations for future research/patent, and their application in microbial pesticide bioremediation.

S. no.	Recommendation	Description	Novelty	Application
1	Genetically engineered microbial strains for multiple pesticide degradation	Engineered microbes expressing multiple degradative enzymes for the rapid breakdown of diverse pesticide groups	Single microbe degrades multiple pesticide classes	Agricultural soil remediation
2	Synthetic microbial consortia for enhanced in-situ pesticide bioremediation	Designed microbial consortia with synergistic metabolic pathways	Metabolic cross-feeding and stability under field conditions	In-situ soil bioremediation
3	CRISPR-based genome editing for enhancing pesticide degradation	CRISPR toolkit for targeted insertion of pesticide-degrading gene clusters	Precision genome engineering	Microbial biotechnology
4	Immobilised enzyme nanocomposite for soil pesticide detoxification	Pesticide-degrading enzymes immobilised on biochar or nanoparticles	Increased enzyme stability and reusability	Soil detoxification
5	Smart biosensor-integrated microbial bioremediation system	Engineered microbes detect pesticide residues and activate degradation pathways	Real-time sensing and response	Precision agriculture
6	Rhizosphere-targeted plant growth-promoting bioremediator microbes	PGPR engineered to degrade pesticides and promote plant growth	Dual remediation and growth promotion	Sustainable agriculture
7	Omics-driven discovery for pesticide-degrading microbial genes	AI-assisted metagenomic screening of soil microbes	Rapid identification of novel degradative genes	Biotechnological research and development
8	Slow-release bioformulation for microbial pesticide degradation	Encapsulated microbes with controlled nutrient release	Extended microbial survival and activity	Field-scale remediation
9	Bioaugmentation kit for pesticide-contaminated agricultural soils	Integrated kit containing microbes, enzymes, and soil conditioners	Complete soil restoration solution	Commercial agriculture
10	Engineered microbial electron donor pathway for persistent pesticide mineralization	Modified electron transport systems to enhance pesticide metabolism	Improved mineralisation efficiency	Environmental biotechnology
11	Consortium-based degradation of organophosphate and neonicotinoid pesticides	Specialised microbial consortium targeting high-toxicity pesticides	Targeted pesticide specificity	Regulatory-compliant remediation
12	Soil-responsive gene expression system for pesticide degradation	Microbial genes activated by soil pH, moisture, or pesticide concentration	Environment-triggered degradation	Smart bioremediation

## 8 Conclusions and future prospects

Microorganisms cause breakdown of the pesticide residues in soil, are inexpensive, environment friendly and do not cause secondary pollution. But their slow rate of pesticide degradation may affect the practicability and efficiency. Research on microbe-assisted degradation of pesticides has been largely conducted. Many pesticide-degrading microbial strains have been identified; however, the practical application of microbial bioremediation is restricted. The key challenges involving microbial degradation of pesticides include the development of highly efficient pesticide-degrading GEMs, cultivation of microbial consortia and the quantitative research on pesticide biodegradation models. Recently, with the development of genetic engineering and molecular biology, on one hand, scientists have started shifting to the construction of efficient engineered bacteria through gene recombination techniques; on the other hand, they have altered the enzyme-producing gene to construct vectors that could express the attributes of degrading pesticides. The future prospects of bioremediation of pesticides are highly promising with the integration of advanced technologies such as synthetic biology, genetic engineering, and artificial intelligence. These innovations are enabling the development of genetically modified microorganisms and plants with enhanced capabilities to degrade persistent pesticide residues more efficiently and selectively. Enzyme engineering and nanotechnology are further improving the stability and delivery of biocatalysts in contaminated environments. Additionally, AI-driven monitoring systems and biosensors are being employed to track bioremediation progress in real-time, optimizing treatment strategies. These advancements not only enhance the efficiency and scalability of bioremediation processes but also support sustainable agricultural practices and environmental restoration.
